# Fibrous epulis of unusual dimensions: case report

**DOI:** 10.11604/pamj.2026.53.56.50151

**Published:** 2026-02-04

**Authors:** Hanane Ammar Boudjelal, Selma Maouene, Rym Bareche, Souad Meddah

**Affiliations:** 1 CHU Blida, University Blida 1, Blida, Algeria

**Keywords:** Epulis, giant epulis, surgery, Algeria, case report

## Abstract

Epulis is a benign inflammatory gingival pseudotumor resulting from chronic irritation or hormonal variations, but neglect, often linked to socioeconomic factors and delayed access to care common in African settings, can lead to massive overgrowth and functional impairment. We report the case of a 56-year-old Algerian patient who presented with an unusually large epulis (8.5 cm x 6 cm x 4 cm). This volume complicated the differential diagnosis and posed an operative challenge in a resource-limited setting. Successful surgical excision was performed, leading to an excellent prognosis. This report underscores the importance of early intervention and highlights the difficulties of managing extensive oral lesions in constrained healthcare environments, serving as a crucial reminder for clinicians.

## Introduction

The term “epulis”, derived from the Greek meaning “on the gum”, refers to an inflammatory pseudotumor of the gingiva. It has no malignant degenerative potential but does have a recurrent tendency [1]. Epulis manifests as a gingival overgrowth resulting from chronic local irritation or hormonal fluctuations [2]. It is typically located on the vestibular side of the incisor-canine region, although it is more rarely found in the molar area [3]. The epulis is often sessile, sometimes pedunculated, and typically presents with an elastic consistency [4]. This inflammatory process is commonly seen in cases of chronic gingivitis of local etiology, in pregnant women, or in patients with certain hematological disorders [4]. Current literature typically classifies epulis into three main histological types: fibrous epulis, granulomatous epulis, and peripheral giant cell granuloma (often referred to as giant cell epulis) [5].

While diagnosis and surgical management are usually uncomplicated [6], as the tumor volume is typically moderate, untreated epulis can grow to a considerable size, leading to significant functional discomfort and major aesthetic disfigurement. These advanced complications are particularly observed in the African context, where delays in diagnosis and treatment are frequently linked to poor socioeconomic conditions [6]. The objective of this case report is to present a patient with an unusually large epulis. The sheer volume (8.5 cm x 6 cm x 4 cm) and clinical appearance of this tumor created a diagnostic challenge, and its management was complicated by an inadequately equipped dental surgery department. We report a case of an unusually large fibrous epulis posing diagnostic and operative challenges in a resource-limited setting.

## Patient and observation

**Patient information:** we report the case of Mr H. M., 56 years old, who consulted the Oral Pathology and Surgery Unit of the University Hospital Center (CHU) in Blida, Algeria, for a large, unsightly gingival mass that had been evolving for more than five years. The patient was thin and asthenic and had difficulty breathing and eating. His medical history was unremarkable, and the only attempted treatment was traditional clove therapy.

**Timeline of current episode:** the gingival mass had been evolving for more than five years. The delay in treatment was multifactorial: the patient was incarcerated during this period, subsequently relocated to Libya, and finally, the COVID-19 pandemic prevented his timely return home. This long evolution without definitive treatment led to the large, unsightly mass that bled on contact with antagonistic teeth.

**Figure 1:**
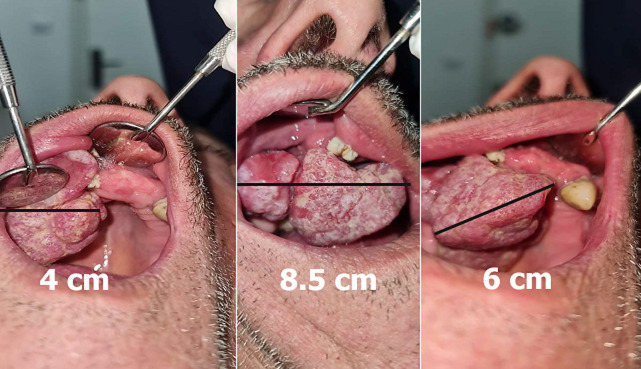
clinical presentation of giant epulis; tumor mass measuring approximately 8.5 cm x 6 cm x 4 cm, filling two-thirds of the upper right vestibule and the right hemi-palate

**Clinical findings:** exobuccal examination revealed facial asymmetry and labial inoclusion, but no cervicofacial adenopathy, sensory disturbance, or pain. Endobuccal examination showed defective oral hygiene, numerous carious, retained roots, and a large mass located in the upper right premolar region. The mass was not adherent to the maxillary bone, was wine-red, covered with a whitish coating in places, and measured approximately 8.5 cm x 6 cm x 4 cm. It was painless, oval, pedunculated, bleeding, with a firm consistency, filling two-thirds of the upper vestibule and the right hemi-palate, with a soft, non-indurated base (Figure 1).

**Diagnostic assessment:** the orthopantomogram showed alveolysis at the base of the tumour pedicle, and the Cone Beam Computed Tomography (CBCT) showed respect for the surrounding structures. The biological work-up, specifically the Complete Blood Count (CBC), was correct, with no abnormalities. Despite the unusual size, a malignant tumor (such as squamous cell carcinoma) was ruled out based on the slow evolution over five years, the mobility of the tumor, the absence of adenopathies, the lack of spontaneous bleeding, and the absence of induration.

**Figure 2:**
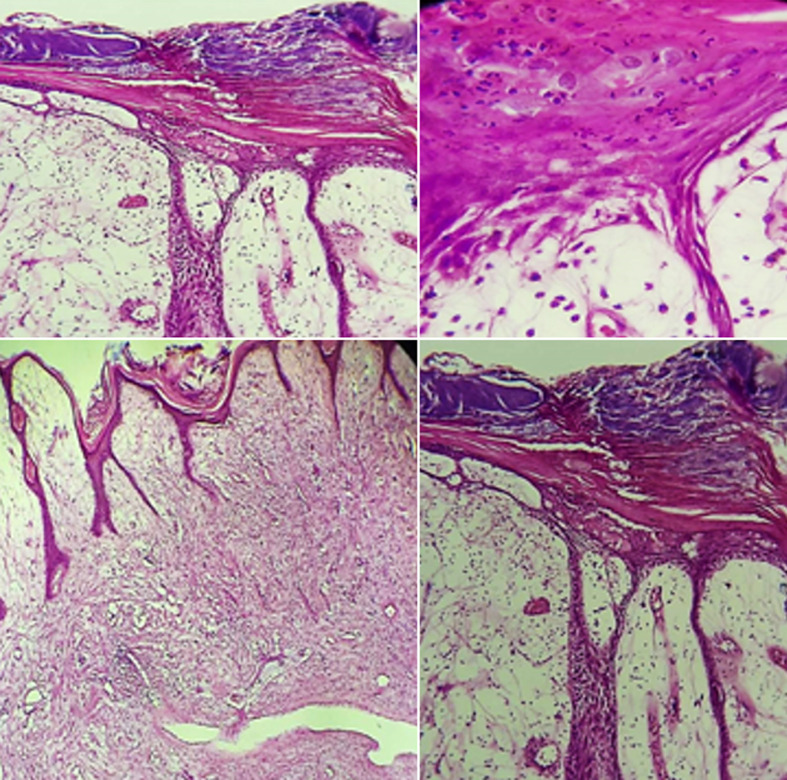
histopathological result showing a nodular lesion, loose fibrous chorion with congested vessels, and fungal superinfection (hematoxylin and eosin)

**Diagnosis:** the surgical specimen was sent for anatomopathological examination. Pathological findings were consistent with fibrous epulis with mycotic superinfection (Figure 2). The prognosis post-excision remains good, provided the pedicle is totally removed and local irritative factors are eliminated.

**Figure 3:**
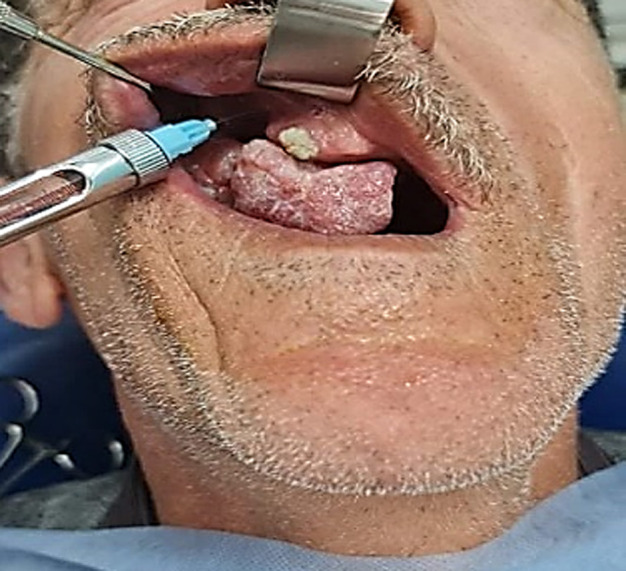
anesthesia with vasoconstrictor (2% mepivacaine)

**Figure 4:**
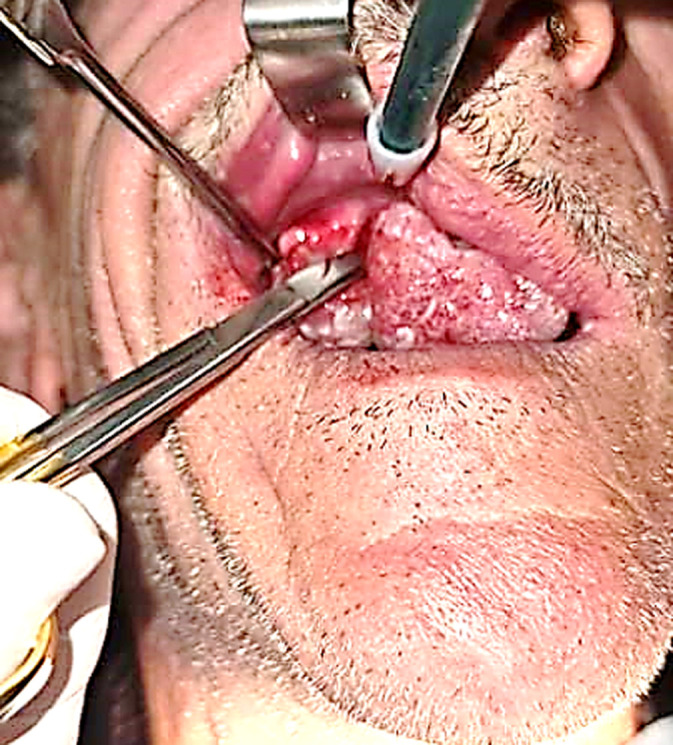
the difficulty of the surgical procedure

**Therapeutic interventions:** surgical excision of the tumor and its pedicle was performed under locoregional anesthesia, supplemented by infiltrations at the base of the tumor using 2% mepivacaine with a vasoconstrictor (Figure 3). The surgical procedure was long and complex due to the operating conditions and the large size of the epulis (Figure 4). Significant intraoperative bleeding was controlled by mechanical compression and sutures, as an electric scalpel or laser was unavailable. A prescription for amoxicillin, paracetamol, and chlorhexidine mouthwash (starting 48 hours after surgery) was recommended.

**Figure 5:**
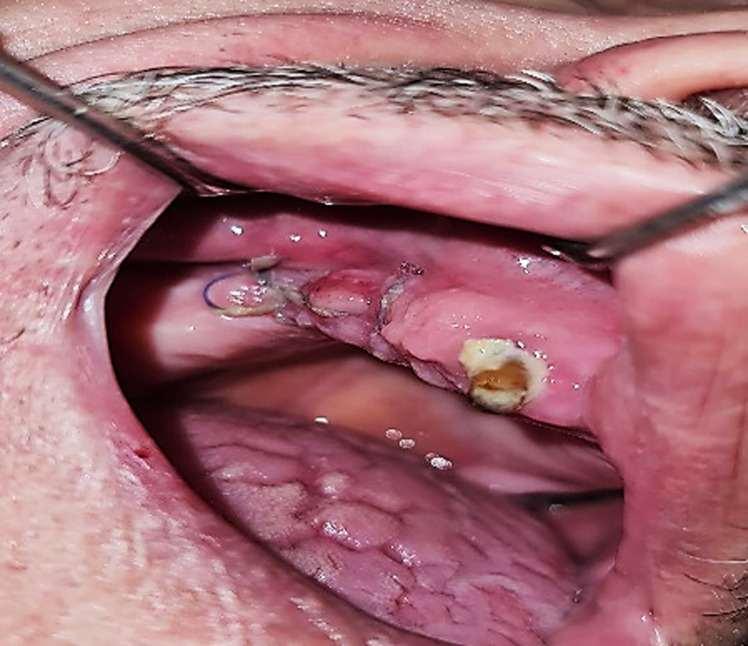
clinical control 7 days after the procedure

**Follow-up and outcome of interventions:** the post-operative course was straightforward. A week later, the patient had regained a normal appearance and a better quality of life (Figure 5). The therapeutic outcome was highly satisfactory, with no recurrence or signs of malignant transformation noted up to the last check-up prior to the publication of this report. The next planned step is dental extractions followed by prosthetic rehabilitation to fully restore function and aesthetics.

**Patient perspective:** the patient expressed immense satisfaction with the outcome, noting an immediate and significant improvement in his breathing, eating, and appearance after the surgery.

**Informed consent:** the patient was fully informed about his condition and the proposed treatment, and provided his written consent for both the surgical procedure and the publication of his case, including the accompanying images.

## Discussion

Epulis is a common, benign hyperplasia of the gingiva, but it rarely reaches the considerable size of the lesion reported in our case. However, in the absence of treatment, the mass can indeed achieve remarkable dimensions, ranging from the size of a walnut to that of an orange. This gigantism is typically attributable to a combination of factors, primarily the significant delay in seeking treatment. In our specific case, the delay was multifactorial: the patient was incarcerated and unable to receive treatment, he subsequently relocated to Libya, and finally, the COVID-19 pandemic prevented his timely return home. Furthermore, the constant, irritating trauma from repeated contact with the opposing lower teeth likely contributed to the excessive growth. This case highlights the constraints of managing extensive oral lesions in a resource-limited setting, which complicated the operative phase. Our therapeutic outcome was highly satisfactory, with no recurrence or signs of malignant transformation noted up to the last check-up prior to the publication of this report. The next judicious step involves scheduling the patient for necessary dental extractions, followed by prosthetic rehabilitation, thereby fully restoring function and aesthetics.

A limited number of comparable cases have been documented in the literature, predominantly within the African context. Our case, measuring approximately 8.5 cm x 6 cm x 4 cm, is comparable to other notable reports of giant epulis. For instance, Fonseca *et al*. [7] reported a case of fibrous epulis in Argentina, which had evolved over 10 years but measured slightly less at 4 cm x 6 cm x 3 cm. Closer in size to ours, Adam *et al*. [3] described a giant epulis in Africa, evolving over 10 years, measuring approximately 9 cm x 6 cm x 4 cm. Larger cases have also been reported, such as that by Adouko-Aka *et al*. [8], which detailed a large epulis in an African woman that had evolved for 3 years, measuring around 11 cm x 6 cm x 4 cm. Similarly, Bengondo *et al*. [9] presented an inflammatory epulis case in an African woman, which had evolved since 2002 and was even larger, measuring 12 cm x 10 cm. A crucial clinical takeaway is that not every bulky mass is necessarily neoplastic. Careful clinical assessment, including palpation of the lesion's base and the search for associated adenopathies, remains vital for preliminary differential diagnosis. The prolonged duration of the lesion's evolution is an important indicator that can help distinguish benign hyperplasia from a rapidly growing malignancy. Despite clinical assumptions, the anatomopathological examination frequently reveals unexpected findings. The overall prognosis for epulis remains good. The definitive absence of recurrence post-excision depends entirely on the total removal of the pedicle and the complete elimination of all contributing local irritating factors.

## Conclusion

This case report underscores that while epulis is a benign lesion, neglect, often compounded by socioeconomic factors and delayed access to care common in African settings, can lead to the development of massive, debilitating lesions. Our patient presented with a giant epulis that posed a significant diagnostic challenge due to its volume and an operative challenge due to the constraints of the clinical setting. The successful management of this case, achieved through complete surgical excision and effective control of bleeding, reaffirms that the prognosis for epulis remains excellent. However, this case serves as a crucial reminder for clinicians: early diagnosis and the complete elimination of local irritating factors are paramount for preventing massive overgrowth and its associated functional and aesthetic complications. Furthermore, it highlights the need for specialized training and resource allocation to manage complex cases in resource-limited environments.
